# Comparative Evaluation of Selected Methods for Assessing Gingival Phenotype

**DOI:** 10.3390/jcm14082669

**Published:** 2025-04-14

**Authors:** Anna Dziewulska, Luiza Czerniawska-Kliman, Agnieszka Droździk, Katarzyna Grocholewicz

**Affiliations:** 1 Department of Interdisciplinary Dentistry, Pomeranian Medical University in Szczecin, 70-111 Szczecin, Poland; luiza.czerniawska.kliman@pum.edu.pl (L.C.-K.); katarzyna.grocholewicz@pum.edu.pl (K.G.); 2 Laboratory of Preclinical Periodontology, Pomeranian Medical University in Szczecin, 70-111 Szczecin, Poland; agnieszka.drozdzik@pum.edu.pl

**Keywords:** gingival phenotype, gingival thickness, periodontal phenotype, assessment tools, ultrasonic measurement

## Abstract

**Background/Objectives:** The diagnostic assessment of soft and hard tissues surrounding the teeth, including gingival phenotype analysis, is critical for clinicians. Since multiple methods for evaluating gingival phenotype have been reported, determining the optimal approach for dental practitioners is essential. This study aimed to evaluate gingival phenotype using visual assessment (VA) and the periodontal probe transparency method (PTM) in the maxillary central incisors to confirm the superiority of the latter. **Methods:** This study included 103 individuals aged 22 to 29 years, all with a healthy periodontium, no history of medications, and no prior treatment affecting the gingiva. Two examiners assessed gingival phenotype using VA and the PTM with color-coded probes. Additionally, direct measurement (DM) with biometric ultrasonography was performed. **Results:** The correlations among VA, the PTM, and DM (Spearman’s rank correlation test) demonstrated robust consistency (r = 0.62–0.76, *p* < 0.001). There was medium to high agreement between VA and DM (r = 0.62–0.74, *p* < 0.001), as well as a medium to strong correlation between VA and the PTM (r = 0.63–0.76, *p* < 0.001), indicating no superiority of the color-coded probe transparency method. **Conclusions:** Both VA and the PTM with a color-coded probe are reliable for identifying the gingival phenotype in the maxillary anterior region when compared to direct biometric measurement.

## 1. Introduction

In the era of esthetic dentistry, awareness of all the factors influencing the final treatment outcome is crucial [1]. The diagnostic assessment of soft and hard tissues surrounding the teeth and implants, including analysis of the gingival phenotype, is a critical aspect for clinicians [2,3]. The term ‘gingival phenotype’ is widely used to describe the bucco-lingual dimension and the width of the keratinized tissues [4,5].

The 2017 World Workshop on the Classification of Periodontal and Peri-Implant Diseases and Conditions recommended adopting the term ‘periodontal phenotype’. However, other reports from this workshop still used the term “periodontal biotype” to analyze the mucogingival status in the natural dentition [6,7].

Periodontal phenotype refers to the combination of gingival phenotype and bone morphotype, while gingival phenotype is determined by gingival thickness (GT) and keratinized tissue width [6]. Evaluation of the gingival or/and periodontal phenotype plays a crucial role not only in maintaining periodontal health but also in the decision-making process for periodontal, restorative, prosthetic, implant, or orthodontic therapy [8]. The anatomy of the periodontium, including gingival thickness, influences its response to various physical and chemical stimuli (e.g., excessive brushing or dental treatment) as well as bacterial invasion [9,10].

A simple and commonly used method of assessing the gingival phenotype is visual evaluation. However, it has been proven that it cannot be considered an objective assessment method, as it accurately diagnoses only about half of the cases and largely depends on the clinician’s experience [11]. Achieving the highest possible objectivity in gingival thickness assessment is crucial. Several techniques can be used to ensure the most reliable results. Although transgingival probing is a simple and commonly used method, it requires local anesthesia. The injection of an anesthetic solution may cause patient discomfort and can temporarily increase local tissue volume [12]. Another commonly used method for assessing gingival thickness is placing a periodontal probe in the gingival sulcus and observing tissue transparency. It is assumed that the probe will be visible when the gingiva is thin (GT ≤ 1 mm) and not visible when the gingiva is thick (GT > 1 mm). This test is highly reproducible, with an 85% agreement between repeated measurements [6,13]. Similarly, ultrasonic devices and cone-beam computed tomography (CBCT) demonstrate high diagnostic accuracy in assessing gingival thickness [14,15,16]. However, CBCT radiation exposure is generally not justified for this specific indication, and ultrasonic devices can be expensive.

Since several methods for evaluating gingival phenotype have been reported, it seems advisable to establish optimal recommendations for dental practitioners. Furthermore, it has been shown that gingival thickness (GT) can vary between individuals due to factors such as age, sex, tooth-related variations, and the impact of the testing method [17]. Knowledge of gingival phenotype properties and their consideration is essential for planning multidisciplinary treatment (periodontal, prosthetic, implant, or orthodontic), enabling the clinician to avoid complications and achieve optimal esthetics [3,18]. On the other hand, there is no consensus in the literature regarding which method of gingival phenotype assessment is the most suitable for everyday practice.

Therefore, the objective of the present study was to evaluate gingival phenotype using visual and periodontal probe transparency methods in the maxillary central incisors. The null hypothesis was that the periodontal probe transparency technique is not superior to the visual assessment method.

## 2. Materials and Methods

This study was conducted in accordance with the Declaration of Helsinki (latest version, 2013), and the protocol was approved by the Bioethics Committee at the Pomeranian Medical University in Szczecin (PUM), with decision reference No. KB-0012/147/17. The study group consisted of 103 participants, aged 22 to 29 years. The post hoc power analysis confirms that the study achieves >90% power for the observed effect sizes, validating its adequacy (see sample size justification in Section 2.2). Study participants were selected based on specific criteria from patients of the Department of Interdisciplinary Dentistry at PUM who reported for preventive treatments. The inclusion criteria were as follows: (-) healthy young individuals aged 20–30 years, (-) no history of systemic diseases, and (-) complete dentition. Exclusion criteria included the following: (-) use of medications known to influence gingival tissue, (-) periodontal disease, (-) fillings and reconstructions located at the gingival line or subgingivally, (-) severe crowding or history of orthodontic treatment, (-) pregnancy or breastfeeding, and (-) smoking or use of other tobacco products.

Participation in the study was voluntary, and each participant provided written informed consent.

### 2.1. Phenotype Evaluation

For the evaluation of the gingival phenotype, three clinical techniques were used. The first method was a visual assessment (VA) in the anterior maxilla region, based on the typical characteristics of the thin and thick phenotype [19]. This was followed by an assessment using the probe transparency method (PTM) with a Colorvue biotype probe (Hu-Friedy, Chicago, IL, USA) [20]. In consecutive order, three probes were inserted into the gingival sulcus of the central upper incisors in a specific sequence: white, green, and blue. The transparency of marginal tissues was observed. The phenotype was then classified as thin, medium, or thick. The visibility of the white tip indicated a thin phenotype. If the white tip was not visible but the green tip was, the phenotype was classified as medium. If the green tip was not visible but the blue tip was, the phenotype was classified as thick. The probe insertion followed the standard technique for probing the gingival sulcus. Both VA and the PTM were conducted by two previously calibrated examiners (A.D.; L.C.-K). Their calibration process, conducted before this study, involved training sessions with a set of 10 volunteers at two week intervals to minimize recall bias while ensuring the stability of gingival conditions. Intra-rater reliability was quantified using Cohen’s kappa. For VA, rater 1 achieved a Cohen’s kappa of 0.89 (95% CI: 0.76–1.00, *p* < 0.001), and rater 2 achieved a kappa of 0.85 (95% CI: 0.71–0.99, *p* < 0.001). For the PTM, rater 1’s kappa was 0.78 (95% CI: 0.63–0.93, *p* < 0.001) for the upper right central incisor and 0.80 (95% CI: 0.66–0.94, *p* < 0.001) for the left, while rater 2’s kappa was 0.75 (95% CI: 0.59–0.91, *p* < 0.001) and 0.77 (95% CI: 0.62–0.92, *p* < 0.001), respectively. The final evaluation involved direct measurement (DM) of gingiva thickness at the mid-buccal aspect of the central incisors. In addition to the keratinized gingiva, the free gingiva was also evaluated as the anatomical structure directly related to the probe transparency method. The measurements were taken using the PIROP ultrasonic biometer (EchoSon, Puławy, Poland) with a fine transducer head (1.7 mm) (device frequency—20 MHz; ultrasonic impulse velocity—1540 m/s; accuracy up to 0.01 mm) [21]. Each tooth was measured in triplicate by a single researcher (A.Dr), an experienced periodontist trained and calibrated with ultrasound measurements [5,21,22,23]. The final thickness value used for analysis was the arithmetic mean of three measurements, each automatically calculated by the device based on ten correctly performed measurements per measurement point.

### 2.2. Statistical Analysis

Sample size justification:

– To detect a moderate Spearman’s correlation (r = 0.3) between gingival assessment methods (e.g., VA vs. PTM) with α = 0.05 and power (1 − β) = 0.8, a minimum of 85 participants were required (correlation analysis),
– For Fleiss’ kappa, detecting substantial agreement (kappa = 0.7) between two raters across two or three categories (e.g., thin/thick or thin/medium/thick) requires approximately 50–60 participants for 80% power at α = 0.05 (inter-rater agreement),
– The Wilcoxon signed-rank test, used to compare gingival parameters between right and left incisors, requires approximately 34 pairs for a medium effect size (*d* = 0.5, α = 0.05, 1 − β = 0.8) (paired comparison).

To estimate the type I error in statistical analysis, a standard significance level of α = 0.05 was adopted. This means that the tolerated risk of rejecting a true null hypothesis (Type I error) is 5%. The Shapiro–Wilk test was applied to assess the normality of the data distribution.

For characterizing the distributions of quantitative variables, basic descriptive statistics were used. Due to deviations from normality in all examined gingival phenotype parameters, the median (Mdn) was chosen as the measure of central tendency. Data dispersion was described using the first (Q1) and third (Q3) quartiles, which are measures of location.

For comparing differences between two dependent groups, where the variable distributions were not normal, the non-parametric Wilcoxon–Mann–Whitney rank-sum test for paired data was applied.

The agreement of categorical variable assessments between two evaluators was examined using Fleiss’ kappa. Correlations between gingival phenotype parameters were estimated using Spearman’s method, and *p*-values were calculated based on the asymptotic approximation of the t-distribution.

To investigate the multifactorial impact of demographic factors on gingival phenotype parameters, while accounting for the repeatability of measurements (left and right side of the upper incisors), a linear mixed model with a robust estimator (RLMER) was used.

The 95% confidence intervals (CI 95%) and *p*-values of the adjusted model were determined using an approximation based on Student’s t-distribution.

The analyses were conducted using the R statistical language (version 4.3.1) [24] on a Windows 10 Pro 64-bit system (build 19,045; Microsoft Corporation, Redmond, WA, USA), with the following packages: lme4 [25], irr [26], robustlmm [27], sjPlot [28], report [29], ggstatsplot [30], and gtsummary [31].

## 3. Results

The results were analyzed for N = 103 patients, 74 females (72.55%) and 28 males (27.45%), with a median age (Mdn) of 25.0 years (minimum of 22.0 years, maximum of 29.0 years).

### 3.1. Visual Assessment of Gingival Phenotype

The results of the visual assessment of gingival phenotype by the two independent raters are shown in Table 1.

The percentage distribution of evaluation between the researchers is quite similar with only a slight difference in the perception of gingival phenotype. The estimated Fleiss’ kappa score of 0.88 for the subjective assessment of gingival phenotypes indicates a very good agreement between the two evaluators. A *z*-value of 8.91 and *p* < 0.001 indicate statistical significance of this agreement.

To address potential gender-related differences in gingival phenotype, the distributions of the visually assessed gingival phenotype was analyzed. Among females, 55.41% (*n* = 41) were classified as exhibiting a thin gingival phenotype, compared to 42.86% (*n* = 12) among males, with thick phenotypes comprising 44.59% (*n* = 33) and 57.14% (*n* = 16), respectively. The difference was not statistically significant (*p* = 0.258, Pearson’s Chi-squared test), indicating that gender does not significantly influence the visually assessed gingival phenotype.

### 3.2. Assessment of Gingival Phenotype with Probe Transparency Method

Table 2 presents the analysis of gingival phenotype distribution using the probe transparency method with color-coded probes conducted by two researchers. The upper right and left central incisors were evaluated as they are important in esthetics and phenotype assessment.

The similarity in assessments between the researchers for the upper right central incisor is high, as reflected in the closely matching percentage values for the respective gingival phenotype categories. Both researchers classify the majority of cases as medium gingival phenotypes (62.14%). The differences in the assessment of the thin (19.42% vs. 20.39%) and thick (18.45% vs. 17.48%) phenotypes were minimal.

Similar relationships were observed for the upper left central incisor. The most frequently identified phenotype was the medium phenotype, and the percentage differences for the thin and thick phenotypes were minimal, indicating no significant discrepancies in evaluation.

The closely matching percentage values for the respective gingival phenotype categories confirms the consistency in gingival phenotype assessments using the PTM conducted by two independent raters. In assessing consistency between raters, the kappa values for overall gingival phenotype differentiation demonstrate substantial concordance, with values of 0.70 for the upper right central incisor and 0.74 for the upper left central incisor. Both results are statistically significant (*p* < 0.001), further supporting the reliability of assessments (Table 3).

The highest observed agreement in the assessment was for the thin gingival phenotype (kappa = 0.82 and 0.73), suggesting that evaluators can distinguish this particularly delicate gingival phenotype with high certainty.

Significant gender differences were observed (*p* = 0.008, Fisher’s exact test). Females predominantly exhibited a medium phenotype (70.27%, N = 52), with fewer cases of thin (17.57%, N = 13) and thick (12.16%, N = 9) phenotypes. In contrast, males showed a more even distribution, with 25% (N = 7) thin, 35.71% (N = 10) thick, and 39.29% (N = 11) medium phenotypes. The higher prevalence of thick phenotypes in males and medium phenotypes in females contributed to this significant difference.

### 3.3. Assessment of the Thickness of Free and Keratinized Gingiva

The distributions of free and keratinized gingiva thickness around the upper central incisors are presented in Table 4.

Neither the free nor keratinized gingiva thickness differed significantly between the right and left central incisors (*p* = 0.589 and *p* = 0.786, respectively), indicating that the gingival phenotype around both incisors is comparable.

### 3.4. Correlation Assessment of Methods for Assessing Gingival Phenotype

The analysis aims to clarify how the visual and color-coded probe transparency of subjective assessments align with measurements of free and keratinized gingiva thickness performed ultrasonically with the use of Pirop. The results are presented in Table 5, with Spearman’s r, 95% CIs, and *p*-values for each pairwise comparison.

The results confirmed consistency across free and keratinized gingiva at both incisors.

The correlations among visual, probe transparency, and direct measurement methods demonstrated robust consistency (r = 0.62–0.76, *p* < 0.001). Moderate to high agreement across the visual method and direct measurement (r = 0.62–0.74, *p* < 0.001) as well as a moderate to strong correlation between visual and probe transparency methods (r = 0.63–0.76, *p* < 0.001) challenge the null hypothesis by showing no superiority of the color-coded probe transparency method.

## 4. Discussion

Clinicians’ understanding of thin and thick periodontal phenotypes, along with their relevance in modern therapies, is essential for achieving optimal treatment outcomes—not only in terms of esthetics but also for long-term soft tissue stability around teeth and implants [2,32,33,34,35,36]. It is well known that positive correlations exist between gingival thickness, keratinized tissue width, bone morphotype, and treatment outcomes in various fields of dentistry [3].

The purpose of our study was to evaluate gingival phenotype using common methods and tools easy to employ in clinical practice. All techniques used in our experiment were non-invasive. Our assessment focused on a young population and maxillary central incisors. The age range was chosen to ensure a relatively homogeneous sample, minimizing potential confounding factors such as age-related periodontal tissue changes. Younger individuals usually exhibit stable gingival status with minimal recession or disease-related changes [37]. Central incisors are among teeth commonly studied in gingival phenotype research due to their particular significance in esthetics. Their susceptibility to recessions justifies their role in periodontal and restorative therapy, as well as in orthodontic treatment planning [38]. Additionally, the visual assessed and translucent probe methods assessed in daily practice appear to be particularly useful in the esthetic zone [13]. In the posterior teeth, evaluating the gingival phenotype before prosthetic reconstructions or implant placement requires adherence to specific recommendations regarding gingival thickness and width, making accurate measurement essential [7].

Many studies assessing maxillary anterior teeth have confirmed a positive correlation between periodontal phenotype, gingival thickness (GT), and keratinized tissue with (KTW) [39,40,41,42,43,44,45,46].

One of the methods used to assess the gingival phenotype, including in our study, is visual assessment. This method is simple and does not require instruments—only knowledge of the characteristics typical of different phenotypes. Although the literature considers the visual assessment of gingival phenotype to be unreliable [11,19], our study noted very good agreement between the two examiners (kappa score of 0.88). This consistency may be attributed to the comparable clinical experience of both investigators, which could have influenced their evaluations of thick and thin gingival phenotypes. While the literature suggests that visual assessment cannot serve as a primary or highly accurate method for determining gingival phenotype [11,19,34], it can still be a useful tool for experienced clinicians.

The second method for phenotype assessment tested in our study was the probe transparency method. Recommended by the World Workshop experts, this method assumes that the probe is visible through the free gingiva in the thin periodontal phenotype (≤1 mm) and not visible in the thick periodontal phenotype (>1 mm) [6].

When determining gingival phenotype using this method, a metal periodontal probe is typically applied; however, an alternative is the phenotype probe, which has a colored tip (white, green, or blue) [34] and was used in our study. Although this method differentiates only two of the three recognized gingival phenotypes [7,11,13,17]—identifying the thin phenotype (corresponding to ‘thin-scalloped’) but not distinguishing between thick-scalloped’ and ‘thick-flat’—it remains highly reliable and reproducible [21,47]. Furthermore, it is one of the most commonly used techniques for analyzing gingival phenotype [6,13,34,47,48,49,50].

The present study revealed similar results obtained by both examiners while applying the probe transparency method, with a kappa score ranging from 0.67 for the medium to 0.82 for the thin gingival phenotype. This suggests that the method has a high degree of repeatability and can be a reliable technique for assessing gingival phenotype around teeth and implants [51,52]. The highest observed agreement was for the thin gingival phenotype, indicating that evaluators were able to distinguish this particularly delicate phenotype with high certainty. Although often considered the gold-standard [53], some studies have shown that the probe transparency method is not entirely accurate in categorizing gingival phenotypes unless the thickness is below 0.6 mm or exceeds 1.2 mm [11,47,54].

The results of the phenotype assessment using two assessors in our study were compared with biometric measurements. For this purpose, an ultrasonic biometer was used. Its ultrasonic transducer, specially designed for measurement in the oral cavity, has a head with a diameter of 1.7 mm, reducing the difficulty in maintaining proper positioning [55]. According to the literature, ultrasonic measurement is as precise as a digital vernier caliper and more accurate and faster than invasive methods such as transgingival probing [21,56,57]. Some studies indicate that ultrasonic devices are the least invasive method for measuring gingival thickness (GT) [47,58]. The validity and reliability of the ultrasonic method have been confirmed to be excellent [14], especially in the hands of a trained clinician [21,26,50,51,52,53,54]. Therefore, biometric measurements were used in our study to compare the tested methods, as they are more suitable for research purposes rather than for routine clinical use due to its cost-effectiveness concerns [12,47].

The analysis of the correlations among the visual, probe transparency, and direct measurement methods in our study demonstrated close agreement (Spearman’s r from 0.62 to 0.76, *p* < 0.001). Moderate to high agreement was observed between the visual method and direct measurement (Spearman’s r from 0.62 to 0.74, *p* < 0.001), as well as a moderate to strong correlation between the visual and probe transparency methods (Spearman’s r from 0.63 to 0.76, *p* < 0.001). These findings challenge the null hypothesis by demonstrating no clear superiority of the color-coded probe transparency method.

However, our findings should be interpreted with caution due to the following limitations:

– The sample was drawn from a single department, which may introduce bias.
– Central incisors cannot represent all teeth.
– We did not consider tooth position, its relation to the alveolar process, overjet, or overbite, all of which influence gingival phenotype.
– We did not account for gingival pigmentation, which may affect the outcome of the probe transparency technique.

Future research should focus on developing a simple, quick, reliable, and cost-effective phenotype assessment method for everyday clinical practice. Additionally, studies on a large, age-diverse population should not only focus on natural dentition but also consider soft tissue around dental implants. Once a consensus in phenotype detection for both teeth and implants is reached through validated diagnostic methods, clinical protocols can be developed to improve esthetics and function after treatment while minimizing the risk of complications.

## 5. Conclusions

Within the limitation of our study, it can be concluded that both visual assessment (VA) and the probe transparency method (PTM) with a color-coded probe serve as reliable tools for periodontal phenotype assessment in the maxillary anterior region when compared to direct biometric measurement. The key findings of this study—very good agreement between the two examiners, as well as high agreement across the tested methods—confirm their clinical applicability and implications for interdisciplinary treatment planning.

## Figures and Tables

**Table 1:** Distribution of visual assessment (VA) of gingival phenotype by rater.

Phenotype	Rater 1, N = 103 (%)	Rater 2, N = 103 (%)
thin	54 (52.43%)	53 (51.96%)
thick	49 (47.57%)	50 (48.04%)

N—sample size.

**Table 2:** The distribution of gingival phenotype assessed by using the probe transparency method (PTM) in the area of the upper central incisors between raters.

	Rater 1, N = 103 (%)	Rater 2, N = 103 (%)
Upper right central incisor		
thin	20 (19.42%)	21 (20.39%)
medium	64 (62.14%)	64 (62.14%)
thick	19 (18.45%)	18 (17.48%)
Upper left central incisor		
thin	17 (16.67%)	18 (17.48%)
medium	70 (68.63%)	69 (67.01%)
thick	15 (14.71%)	16 (15.46%)

N—sample size.

**Table 3:** Consistency of gingival phenotype assessments with probe transparency method (PTM), N = 103.

Category	Upper Right Central Incisor		Upper Left Central Incisor	
	Kappa	*p*	Kappa	*p*
Overall, including the following:	0.70	<0.001	0.74	<0.001
thin	0.73	<0.001	0.82	<0.001
medium	0.67	<0.001	0.70	<0.001
thick	0.70	<0.001	0.71	<0.001

Kappa—Fleiss’ kappa; *p*—*p*-value.

**Table 4:** The distributions of free and keratinized gingiva thickness evaluated using direct ultrasonic measurement, categorized by the side of the central upper incisor.

Characteristic	N	Central Upper Incisor		*p* ^b^
		Right, N = 103 ^a^	Left, N = 103 ^a^	
Thickness of free gingiva (mm)	103	0.96 (0.68. 1.13)	0.93 (0.72. 1.09)	0.589
Keratinized gingival thickness (mm)	103	0.97 (0.80. 1.09)	0.94 (0.81. 1.09)	0.786

^a^ Mdn—median (Q1—first quartile (25%), Q3—third quartile); ^b^ Wilcoxon signed-rank test for paired data; N—sample size; *p*—*p*-value.

**Table 5:** The correlation of methods for assessing gingival phenotype.

Variable	Comparison	Spearman’s r	95% CI	*p*-Value
Upper right central incisor				
Free gingiva	VA vs. PTM	0.71	0.61–0.80	<0.001
	VA vs. DM	0.62	0.50–0.72	<0.001
	PTM vs. DM	0.67	0.55–0.76	<0.001
Keratinized gingiva	VA vs. PTM	0.69	0.58–0.78	<0.001
	VA vs. DM	0.64	0.52–0.74	<0.001
	PTM vs. DM	0.66	0.54–0.76	<0.001
Upper left central incisor				
free gingiva	VA vs. PTM	0.76	0.66–0.85	<0.001
	VA vs. DM	0.74	0.63–0.84	<0.001
	PTM vs. DM	0.75	0.66–0.84	<0.001
Keratinized gingiva	VA vs. PTM	0.63	0.51–0.73	<0.001
	VA vs. DM	0.63	0.52–0.73	<0.001
	PTM vs. DM	0.69	0.59–0.78	<0.001

CI = confidence interval. *p*-values < 0.05 indicate statistical significance.

## Data Availability

The data presented in this study are available on request from the corresponding author due to privacy and ethical reasons.
